# Small and Long Non-coding RNAs as Functional Regulators of Bone Homeostasis, Acting Alone or Cooperatively

**DOI:** 10.1016/j.omtn.2020.07.017

**Published:** 2020-07-15

**Authors:** Mateusz Sikora, Krzysztof Marycz, Agnieszka Smieszek

**Affiliations:** 1 Department of Experimental Biology, Faculty of Biology and Animal Science, University of Environmental and Life Sciences Wroclaw, Norwida 27B Street, 50-375 Wroclaw, Poland; 2 International Institute of Translational Medicine, Jesionowa 11 Street, 55-124 Malin, Poland; 3 Collegium Medicum, Institute of Medical Science, Cardinal Stefan Wyszynski University (UKSW), Wóycickiego 1/3, 01-938 Warsaw, Poland

## Abstract

Emerging knowledge indicates that non-coding RNAs, including microRNAs (miRNAs) and long-noncoding RNAs (lncRNAs), have a pivotal role in bone development and the pathogenesis of bone-related disorders. Most recently, miRNAs have started to be regarded as potential biomarkers or targets for various sets of diseases, while lncRNAs have gained attention as a new layer of gene expression control acting through versatile interactions, also with miRNAs. The rapid development of RNA sequencing techniques based on next-generation sequencing (NGS) gives us better insight into molecular pathways regulated by the miRNA-lncRNA network. In this review, we summarize the current knowledge related to the function of miRNAs and lncRNAs as regulators of genes that are crucial for proper bone metabolism and homeostasis. We have characterized important non-coding RNAs and their expression signatures, in relationship to bone. Analysis of the biological function of miRNAs and lncRNAs, as well as their network, will pave the way for a better understanding of the pathogenesis of various bone disorders. We also think that this knowledge may lead to the development of innovative diagnostic tools and therapeutic approaches for bone-related disorders.

## Graphical Abstract

**Figure:**
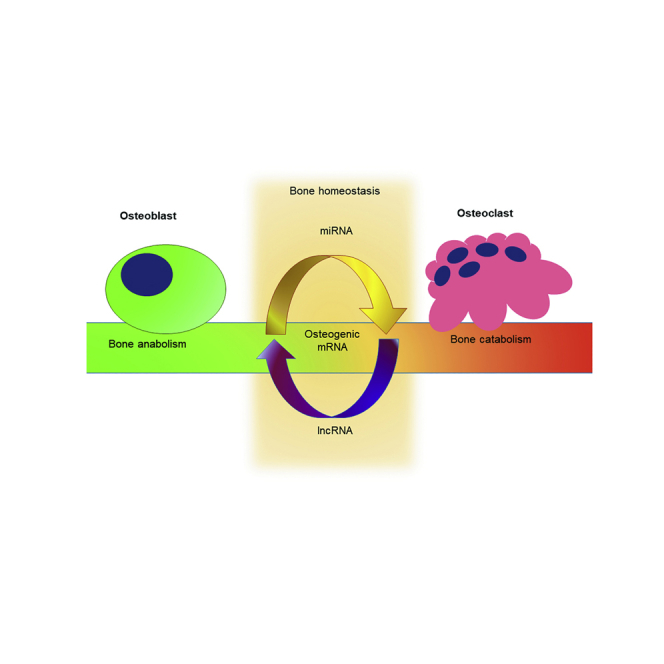

## Teaser

Non-coding RNAs have started to gain attention as molecular regulators of bone homeostasis and potential biomarkers of bone turnover. The molecular network between microRNAs (miRNAs) and long non-coding RNAs (lncRNA) is an essential modulator of osteogenic gene expression. Current efforts are aimed at understanding the interplay between miRNA and lncRNA in order to provide promising molecular targets and therapeutic molecules for bone healing.

## Main Text

Bone disabilities are prevalent across the life-course and affect the skeletal system. Predominantly, they can be a result of degenerative conditions, trauma, developing cancers, and infections associated with inflammations. Consequently, modern strategies of bone repair have been intensively developed, especially in terms of osteogenic and bone turnover molecular markers.^1^ The proper recognition of molecular mechanisms engaged in the development of metabolic bone diseases and bone cancers is crucial for development of new treatment options. Many are commonly available, including palbociclib, which is aimed at silencing cyclin-dependent kinase (CDK)4 and CDK6 in breast cancer treatment.^2^ However, numerous treatment methods based on molecular pathways still remain under investigation.

Recently, non-coding RNAs, including small non-coding RNAs (i.e., microRNAs [miRNAs]) and long non-coding RNAs (lncRNAs) have gained recognition as another epigenetic layer of regulation in many tissues, including bone. The biomarker validity of both circulating and endogenous non-protein-coding RNAs is of great importance in bone-related diseases and disorders, such as osteoporosis (OP), rheumatoid arthritis (RA), or osteosarcoma (OS).^3^ miRNAs, as well as lncRNAs, are considered to be important regulators of gene expression. Their ability to function as key players in the development of pathological conditions has been discussed for many years. Therefore, the high degree of involvement of non-coding RNAs in transcriptomic activity is closely related to their use as prognostic and diagnostic factors. Despite recent advances in non-coding RNA studies, the biology of these molecules still remains unclear.^4^ High-throughput technologies, e.g., next-generation sequencing (NGS), have led to the expansion of the understanding of the non-coding RNA world. The proper combination of multidisciplinary and interdisciplinary approaches has proven essential in revealing the complexity of the non-coding RNAs network and to support establishing the importance of their regulatory existence.^5^

The aim of this review is to highlight the connection between miRNAs and lncRNAs, which is clearly observed in the course of osteogenesis. Both miRNA and lncRNA have potential as diagnostic and prognostic markers, and thus growing evidence indicates that they can be considered as therapeutic targets in bone-related diseases. The clinical value and roles of non-coding RNAs are increasing, especially in the field of regenerative medicine. In this review, we emphasize emerging potential of non-coding RNAs as markers of bone turnover in commonly occurring bone-related diseases, such as osteoporosis, RA, and bone cancers (OS). In this review, we describe the role of several miRNAs, classified as osteo-miRs. The review summarizes the current knowledge about the function of *miR-21-5p* (*miR-21*) *miR-124-3p* (*miR-124*), *miR-203-3p* (*miR-203*), and *miR-223-3p* (*miR*-233) in bone biology. Moreover, we provide information about the role of several lncRNAs closely connected with bone metabolism. We described the function of differentiation antagonizing non-protein-coding RNA (*DANCR*), taurine upregulated gene 1 (*TUG1*), metastasis-associated lung adenocarcinoma transcript 1 (*MALAT1*), and HOX transcript antisense intergenic RNA (*HOTAIR*) in order to indicate their actual therapeutic potential. Non-coding RNAs presented in the review are widely considered to be important markers affecting proper bone homeostasis in the described bone diseases.

Additionally, we have presented current information regarding crosstalk between selected miRNAs and lncRNAs, indicating their important role as regulators of molecular pathways during osteogenesis.

As of the time of this writing, only 27 original studies were identified by a literature search in PubMed’s collection of articles that are related to non-coding RNA networks. The combined search terms were “lncRNA” and “miRNA” and “network” and “bone.” To date, no review study has been published that referred to all of the assumed requirements. For this reason, in this review we decided to place an emphasis on the connections linking non-coding RNAs and putative mRNAs, which are essential for proper bone metabolism. In this review, we have pointed out non-coding RNAs that have gained attention as potential biomarkers of bone development or future therapeutic targets.

### Biological Functions of Non-coding RNAs

#### Biology of miRNA

miRNAs (miRs) are small endogenous non-coding molecules, 19–25 nt in size. Their main function is regulation of post-transcriptional silencing of target genes. A single miRNA can target many mRNAs and influence the expression of hundreds of genes involved in functional signaling pathways essential for the survival, proliferation, or differentiation of cells.^6^ For example, *miR-15a* and *miR-16-1* are considered to be crucial molecules that inhibit cyclin D expression and suppress OS progression.^7^ It was proven that miRNAs regulate the expression not only of mRNA, but also other non-coding RNAs such as lncRNAs. For instance, it has been demonstrated that *miR-125b* expression is negatively correlated with *MALAT1* expression. Furthermore, the *HCA1* transcript level is highly associated with *miR-1* activity, due to a binding region for *miR-1* in the *HCA1* structure.^8^ The well-known mechanism of miRNA action reduces the expression of targeted mRNAs. The miRNAs, due to their complementarity to the sequence of mRNAs, are able to interact with them and regulate their expression. The most well-described mechanism of miRNA-mRNA interaction is via the 3′ UTR region of target mRNAs, resulting in suppressed expression. Moreover, the interaction of miRNAs with other regions, such as the 5′ UTR, coding sequence, and gene promoters, has also been noted.^9^ It has been shown that miRNA interaction with the promoter region may induce transcription in certain conditions.^10^ The described modes of miRNA-mRNA interactions are still being studied in order to depict their functional significance.

Single miRNAs may have a plethora of effects and can be regarded as pleiotropic molecules. For example, *miR-21* is a regulator of many genes, inducing tumorigenesis, and thus is often considered to be a major onco-miR. At the same time, it is an important molecule regulating pro-osteogenic genes, facilitating the proliferation and differentiation of osteoblast precursors.^11^^,^^12^ The levels of miRNAs are regulated by mechanisms similar to other RNAs such as transcriptional activation and inhibition, epigenetic repression, and degradation. It has been proven that a miRNA expression profile changes, depending on the physiological state of the organism.^13^ Therefore, they are often used as diagnostic markers, alone or with other molecules, including mRNAs. For instance, tremendous metastatic potential of tumors is associated with a high *miR-21* level and correlates with elevated expression of cyclin D, which plays a key role during cancer cell proliferation. It is therefore a valuable prognostic marker associated with a poor prognosis.^7^

In the canonical miRNA biogenesis pathway, miRNA genes are transcribed by Pol II (polymerase II). The long primary transcripts have a local hairpin structure where the miRNA sequence is embedded. In humans, many canonical miRNAs are encoded within introns of coding and non-coding transcripts. However, some miRNAs are encoded within exonic regions.^9^^,^^14^ After transcription, the primary miRNA (pri-miRNA) is maturated by a nuclear microprocessor consisting of an RNase III Drosha and a DGCR8 cofactor. The essential role of miRNA during an organism’s development has been proven, because deficiency of RNase III Drosha/DGCR8 causes lethality in early embryogenesis.^14^ Following Drosha processing, the created pre-miRNA is exported to the cytoplasm through exportin 5 (XPO5) and forms a transcript complex with guanosine triphosphate (GTP)-binding nuclear protein. In the cytoplasm, pre-miRNA is cleaved by Dicer (RNase III endonuclease). This processing involves the removal of the terminal loop, resulting in small RNA duplex creation. Furthermore, the duplex generated by Dicer is loaded onto a protein called AGO (Argonuate). This complex is known as RISC (RNA-induced silencing complex).^9^^,^^14^ Meanwhile, the passenger strand of the miRNA duplex is degraded and an active single guide strand recognizes the mRNA transcript. This specific binding inhibits translation and promotes the degradation of mRNA targets.^15^ The biogenesis of miRNA may also occur by alternate, non-canonical pathways, including both DROSHA-independent and DICER independent processes; however, the role of non-canonical miRNAs in bone biology is not well described.^16^

Furthermore, miRNAs are released into the bloodstream, in part through active secretion. About 90% of extracellular miRNAs are bound to AGO proteins, and only 10% are packed in apoptotic bodies, exosomes, or high-density lipoprotein (HDL).^17^ Circulating miRNAs actively regulate bone metabolism and thus can be regarded as “fingerprints” for many bone-related diseases, such as osteoporosis or bone tumors.^17^^,^^18^ Circulating miRNAs are effectively detectable in liquid biopsies, including plasma, serum, and urine. These are minimal or even non-invasive sources of biomarkers with great potential for diagnostics and clinic application, because circulating miRNAs are more stable in fluids. However, the levels of circulating miRNA are lower than those found in tissues and cells. Nevertheless, liquid biopsies as a source stable and reliable markers gives a huge advantage in terms of treatment of bone diseases in which biopsy may be problematic.^19^

Given the fact that miRNAs are engaged in the progression of many human diseases, they are a significant potential diagnostic and prognostic factor. Moreover, recent technological advances have contributed to the significant growth of miRNA validity and enhanced strategies of miRNA-dependent therapies.

#### Biology of lncRNAs

lncRNAs are a family of long (200–100,000 nt long) transcripts with very low protein-coding potential and a structure similar to mRNA. However, some lncRNAs can encode short peptides. Transcripts derived from lncRNAs constitute 4%–9% of the mammalian genome, in comparison to protein-coding sequences, which are 1% of the genome.^4^ Recent studies have shown that lncRNAs play a crucial role in developmental processes and that they are responsible for nuclear chromatin structure regulation as well as gene expression. Nonetheless, there are also opinions that lncRNAs are transcriptional noise and a by-product of RNA Pol II transcription.^4^ The expressed amount of different lncRNAs varies in different tissues, indicating that they are tissue-specific molecules, similarly to miRNAs. Furthermore, the general amount of expressed lncRNAs in every cell is lower than the quantity of miRNA transcripts. Moreover, interspecies homology of lncRNA sequences is relatively low compared to miRNAs. Nevertheless, a certain degree of conservation in the promoter region and exon area of lncRNAs is observed, which suggests that these molecules are biologically significant.^4^ lncRNAs are quite often abundantly expressed in a controlled manner in cells, which have open and active chromatin, such as stem or progenitor cells.^20^ lncRNAs have been found to be highly expressed in embryonic stem cells, regulating their renewal, differentiation, and pluripotent state. Moreover, lncRNAs are expressed in a controlled manner, similarly to morphogens, and have been indicated as crucial regulators of various developmental pathways during organogenesis.^20^ For example, it was shown that lncRNAs are a vital regulator of osteogenesis induced in a progenitor derived from a mesenchymal lineage.^21^ However, lncRNAs are also identified as essential regulators of many pathological processes such as osteoporosis or osteoarthritis.^22^ Accumulating evidence has demonstrated that dysregulation of lncRNAs is an important component in the gene regulatory networks during the development and progression of cancer. Thus, lncRNAs are being considered as potential targets in terms of cancer treatment, or biomarkers with diagnostic and prognostic potential.^23^

The mechanism of lncRNA action is highly complex and has not yet been fully understood, due to initial knowledge about this type of RNA. It is thought that lncRNAs affect mRNA functionality through various pathways. It was proved that lncRNAs participate in gene expression patterns at the transcriptional and post-transcriptional levels.^24^ First, lncRNAs can recruit a chromatin remodeling complex to specific sites and regulate the expression process. Second, lncRNAs can regulate transcriptional expression through blocking the promoter region, interacting with RNA-binding proteins, or regulating the activity of transcription factors. Moreover, lncRNAs can form double-stranded RNA complexes with mRNA at the post-transcriptional level.^4^

**Figure 1:**
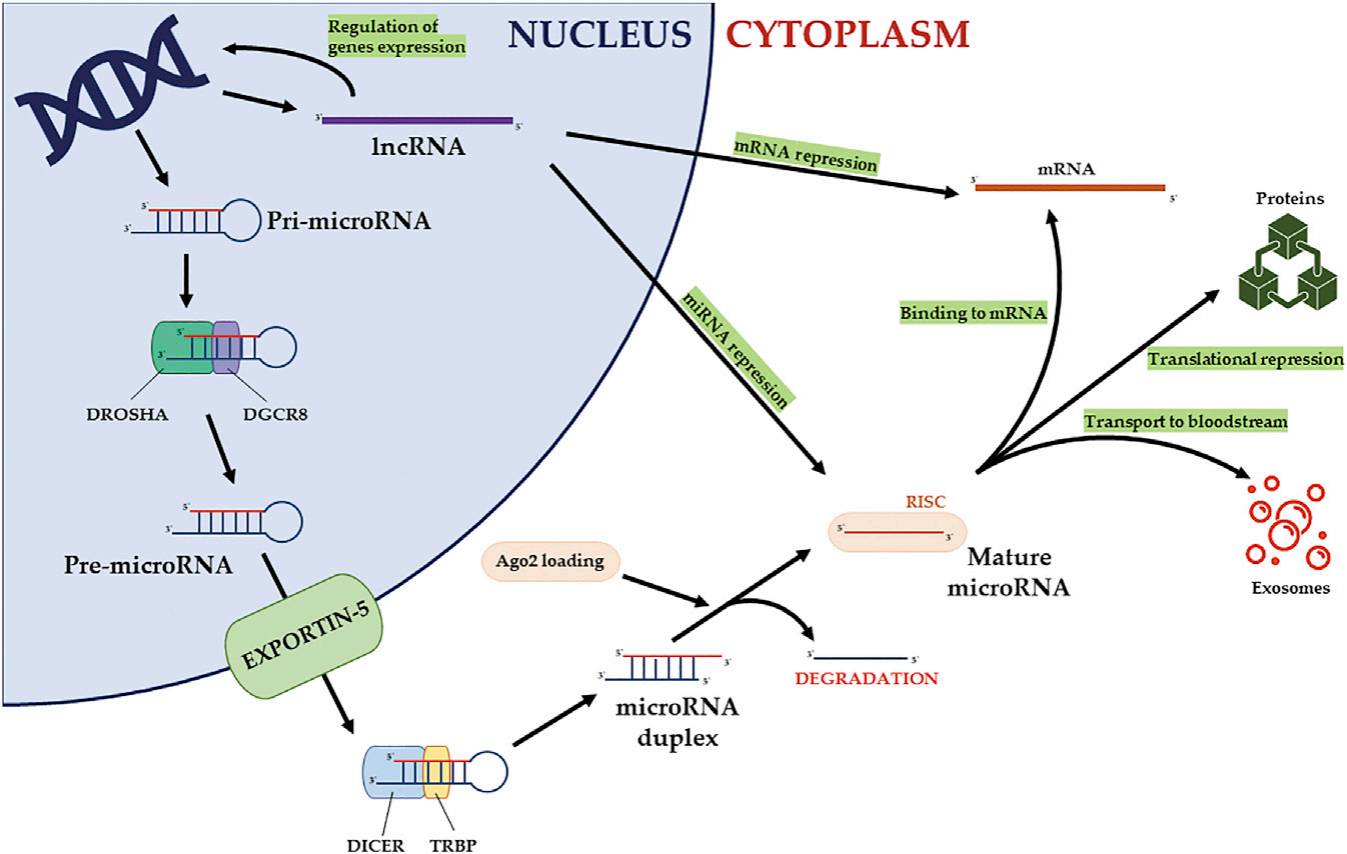
The Biogenesis and Mechanisms of Action of Non-protein-Coding RNAs (miRNAs and lncRNAs) The schema of miRNA expression shows the canonical pathway of miRNA biogenesis. Additionally, there is demonstrated crosstalk between miRNA and lncRNA that ensures the variable concentration of non-coding RNAs.

Many efforts are being made in terms of clarification of the biological function of lncRNAs, both as a regulator of essential developmental pathways and as regulators of tumorigenesis. In this review, we summarize the knowledge regarding the function of lncRNAs as pro-osteogenic factors (Figure 1).

### The Role of Selected Non-coding RNAs in Bone Biology and Disease Pathogenesis

The maintenance of bone homeostasis is preserved by the activity of non-protein-coding RNAs. Both miRNAs and lncRNAs are extremely important factors that lead to proper cell functionality by affecting the expression of crucial genes.^13^^,^^23^ However, the supportive roles of several molecules have been explored more extensively. Apparently, this is a result of certain dependencies that link these non-coding RNAs into clear and well-established signaling pathways essential for the maintenance of homeostasis. Moreover, researchers have started to pay attention to the specific crosstalk between lncRNAs, miRNAs, and targeted mRNAs. More recent studies have started to show the great importance of specific non-coding RNAs and their relationship with regulated genes.^23^^,^^24^ Additionally, it has been proven that miRNAs significantly modulate mRNA expression, while lncRNAs are responsible for the functioning of both miRNAs and mRNAs.

**Figure 2:**
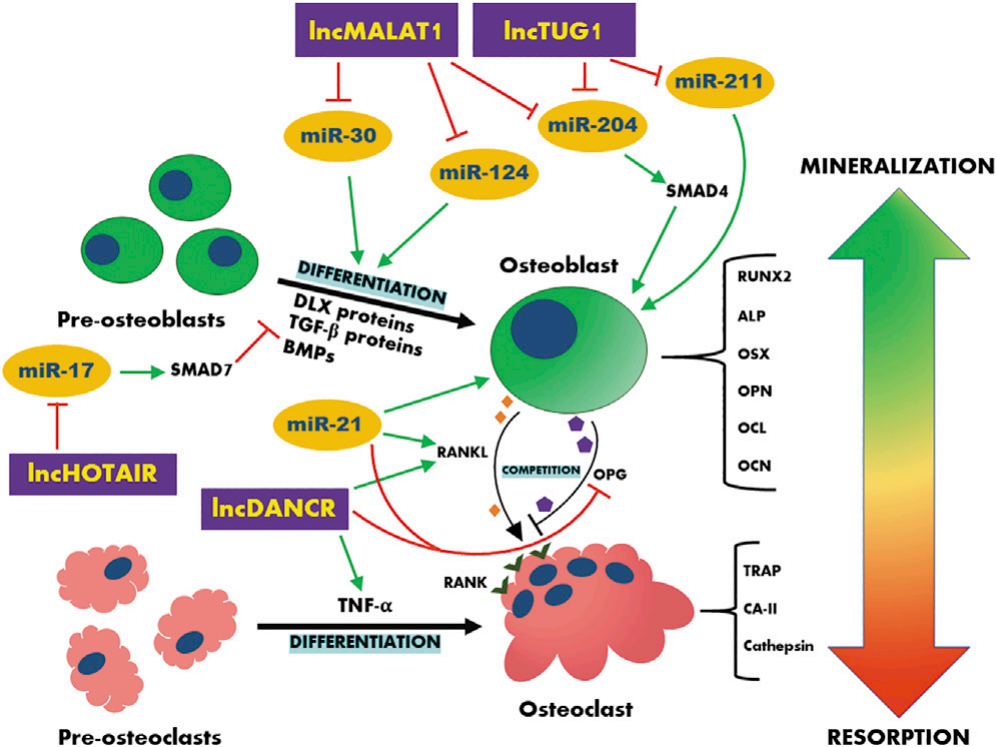
The Crosstalk between Selected lncRNAs, MicroRNAs, and Their Targets in Bone Attention was paid to cell differentiation and coupling mechanism between osteoblasts and osteoclasts. Moreover, the presented signaling pathways place an emphasis on the regulating mechanisms between lncRNAs, miRNAs, and targeted mRNAs. The examined pathways indicate the close relationships between all of the presented molecules in maintaining bone homeostasis. Non-coding RNAs regulate differentiation of progenitor cells and promote survival of both osteoblasts and osteoclasts. Green arrows indicate a positive impact (elevated expression), red arrows indicate a negative connection (reduced expression).

The essential functions of *miR-21* and *miR-124* in bone tissue turnover were previously described in detail.^25^^,^^26^ This pair of miRNAs works in an opposite way and takes part in bone-dependent disease progression. *miR-21* is known from its engagement in the process of osteogenic differentiation of bone marrow-derived mesenchymal stem cells (BMSCs), as well as maintaining proper bone remodeling. However, it can also contribute to the development of bone neoplasms. In contrast, *miR-124* leads to aggressive osteoclast invasion, which leads to active bone resorption and bone metabolism disorders, such as osteopenia or osteoporosis. Furthermore, the potential osteogenesis modulatory abilities of *miR-203* and *miR-223* remain under investigation and need to be clarified in subsequent experimental analyses. This pair of miRNAs actively participates in both processes, i.e., bone mineralization and bone resorption; however, the regulatory roles of these molecules remain unclear. Furthermore, *TUG1*, *MALAT1*, and *HOTAIR* are lncRNAs that are among the important regulators of tumorigenesis.^21^ They are regarded as future molecular targets with extremely high prognostic and diagnostic potential. In addition, the lncRNA *DANCR* is known for its essential engagement into the bone turnover dysregulation that leads to systemic bone disorder development, e.g., osteoporosis (Figure 2).^4^

The mechanisms of action of these molecules strictly refer to bone-related disorders, and for that reason they are broadly described in this review. Data referring to the molecular network of presented molecules is also provided.

#### miR-21

The role of *miR-21* as a molecule regulating osteogenesis has been widely investigated, due to an important connection between *miR-21* expression and development of bone disorders, such as osteoporosis, osteoarthritis, or bone cancers. The role of *miR-21* has been tested using various osteoprogenitor cells. For example, it was shown that *miR-21* promotes the level of osteogenic differentiation and increases matrix mineralization in osteogenic cultures of mouse pre-osteoblasts, i.e., the MC3T3-E1 cell line. The study showed that *Smad7*, which inhibits transforming growth factor β (TGF-β) signaling, is a direct target of *miR-21* in MC3T3-E1 cells. Similarly, it was shown that *miR-21* is crucial for mineralization capability of BMSCs, and that this process is also regulated by the Smad7-Smad1/5/8-runt-related transcription factor 2 (Runx2) pathway. Our recent data showed complex engagement of *miR-21* in the process of osteoblast-osteoclast coupling.^25^ We have confirmed previous studies showing that inhibition of *miR-21* expression in MC3T3-E1 cells causes a decrease in mRNA expression of crucial osteogenic markers, such as osteocalcin (Ocl), osteopontin (Opn), collagen type I, and Runx2. Furthermore, we confirmed that MC3T3-E1 cells with lowered expression of *miR-21* did not support the osteoclastogenesis process, which might be related to the fact that its targets, such as Opn or receptor activator of nuclear factor κB ligand (RANKL), are regulated in a dynamic manner in the process of osteogenesis.^25^

Other studies conducted with the use of MSCs have shown similar close dependencies. In human BMSCs (huBMSCs) the elevated expression of *miR-21* affects the overexpression of typical osteogenic markers, such as *RUNX2* or osteonectin (*OCN*).^27^^,^^28^ Additionally, the key targets of miR-21, including SRY-box 2 (*SOX2*), one of the four genes promoting induced pluripotent stem cells (iPSCs), and sprout homolog 2 (*SPRY2*), negatively regulate the extracellular signal-regulated kinase-mitogen-activated protein kinase pathway.^27^ Furthermore, the overexpression of *miR-21* can repress the expression of interleukin (IL)-6 and IL-8, which are involved in the Wnt signaling pathway. Moreover, Wnt signaling is highly engaged in cell commitment and maintenance of bone homeostasis. Therefore, therapy based on *miR-21* delivery could simultaneously relieve the symptoms of RA.^29^^,^^30^

In contrast, *miR-21* plays a vital role in FLS (fibroblast-like synoviocyte) invasiveness and significantly affects the expression of matrix metalloproteinases. Moreover, the inhibition of *miR-21* activity downregulates the expression of TGF-β and *Smad5* but increases the *Smad7* transcript levels.^30^ Thus, inhibition of *miR-21* could serve as a favorable therapeutic target diminishing FLS metabolic activity and lowering the symptoms of joint diseases. Moreover, *miR-21* seems to be a crucial factor that affects the expression of pro-inflammatory cytokines, both *in vitro* and *in vivo* during periodontitis.^31^

*miR-21* is commonly known as an oncomiR and is significantly overexpressed in many cancers, including OS.^11^ The high level of *miR-21* is correlated with initial metastasis, poor response to neoadjuvant chemotherapy, and reduced overall survival rate.^32^ The expression of *miR-21* is also positively correlated with the presence of lung metastases in OS patients. Therefore, it can serve as a potential biomarker for the early diagnosis of OS and considered to be a future anti-cancer target.^32^^,^^33^

Bearing in mind the significance of *miR-21* in bone homeostasis maintenance, novel kinds of molecular therapy have been developed. *miR-21* can be used not only as diagnostic factor in cancer therapy, but especially as an important therapeutic target in many bone diseases. Modern methods based on targeted *miR-21* inhibition can improve the effectiveness of common anti-cancer therapies and increase the survivability of patients.^34^^,^^35^ Furthermore, the targeted delivery of *miR-21* can produce excellent results in the regeneration of bone fractures.^36^^,^^37^

#### miR-124

*miR-124* is a highly conserved miRNA, and it is overexpressed in many cancers, such as breast cancer, gastric cancer, or glioblastoma.^38^ Other reports have demonstrated that *miR-124* has a strong inhibitory effect on various human neoplasms, such as gliomas, sarcomas, or liver cancers.^39^, ^40^, ^41^ Researchers have found various roles of *miR-124*, e.g., cell cycle arrest, epithelial-to-mesenchymal transition (EMT), cancer stem formation, induction of apoptosis, or even metastasis creation. Therefore, it might be regarded as a good target for designing novel anti-cancer therapeutic strategies.^38^ In bone cancers, *miR-124* is considered to be potential anti-cancer agent for OS treatment, due to suppressing growth and aggressiveness of this cancer cells.^41^^,^^42^ Transfection of *miR-124* significantly decreases integrin expression and inhibits OS growth, proliferation, migration, and formation of metastases. It also attenuates OS resistance to various drugs, such as tunicamycin, by downregulation of *P53* and *Bcl-2* genes.^41^ Additionally, *miR-124* suppresses TGF-β expression in tumor cells.^41^ The inhibition of OS aggressiveness suggests that *miR-124* can be a potential anti-cancer target for OS therapy. Moreover, it was also demonstrated that *miR-124* negatively regulates the process of osteogenesis and proper bone regeneration of BMSCs.^43^ *miR-124* negatively affects osteogenic differentiation of MSCs and *in vivo* bone formation. It acts as an endogenous attenuator of several genes belonging to the homeobox transcription factor gene family, e.g., *Dlx5*, *Dlx3*, and *Dlx2* expression, by binding the 3′ UTRs of these genes.^26^ The members of the DLX gene family are responsible for bone development and the healing of fractures. For this reason, *miR-124* is considered to be an anti-osteogenic molecular marker. In addition, *miR-124* targets *CDK2* (cyclin-dependent kinase 2) and *MPC-1* (monocyte chemotactic protein-1), which are involved in the inflammatory process in RA.^44^ Previous studies have indicated that *miR-124* directly targeted osterix (*Osx*) expression. Osterix is expressed by osteoblasts and is predominantly responsible for bone formation and homeostasis by promotion of osteoblast differentiation and maturation.^45^ Therefore, the therapy based on knockdown of *miR-124* would be the most efficient in treatment of both osteoporosis and RA.

Interestingly, the high concentration of *miR-124* downregulates the expression of glycogen synthase kinase 3β (*GSK-3β*). This molecule is a significant marker leading to inhibited differentiation of ligament fibroblasts into osteoblasts. Hence, high expression of *miR-124* can accelerate the progression of ankylosing spondylitis, which is connected with spastic and spinal joint disabilities, as well as pathological ossification.^46^

#### miR-203

*miR-203* overexpression is primarily related to downregulation of Runx2 expression, the key factor in osteogenesis.^47^ Laxman et al.^48^ proved that overexpression of *miR-203* inhibits osteoblast differentiation, whereas inhibition of *miR-203* stimulates alkaline phosphatase (ALP) activity and bone matrix mineralization. It was also shown that *miR-203* negatively regulates *BMP-2* (bone morphogenetic protein 2) expression by suppressing *Dlx5*, which is one of the key factors in bone repair. However, *miR-203* was also found to be essential in the shift from osteogenic differentiation to adipogenic differentiation of BMSCs in postmenopausal osteoporosis. The transfection of *miR-203* led to elevated expression of osteogenic genes such as *Runx2* or *ALP* in osteoporotic samples.^49^ Furthermore, the *miR-203* level is upregulated in RA-delivered tissues.^50^ Elevated levels of *miR-203* lead to increased secretion of matrix metalloproteinase (MMP)-1 and IL-6 via the nuclear factor κB (NF-κB) pathway and in this way activate the phenotype of synovial fibroblasts in RA.^51^ Hence, *miR-203* plays the role of a pro-inflammatory and joint-destructive factor in RA. *miR-203* is also associated as a strong tumor suppressor. Huang et al.^52^ have shown that transfection of *miR-203* inhibits TGF-β-induced EMT, migration, and invasive ability in non-small-cell lung cancer by targeting *Smad3*. However, it was also shown that *miR-203*, predominantly associated with EMT, was significantly elevated in plasma samples of ovarian cancer patients.^53^ Moreover, *miR-203* acts as a strong tumor suppressor in OS cells, regulating *RUNX2* and *RAB22A* expression.^54^^,^^55^ Moreover, Liu and Feng (2015) indicated anti-tumor properties of *miR-203* in OS cell lines and tissues. *miR-203* targets TANK binding kinase 1 (*TBK1*), which was found to be upregulated in OS samples.^56^ In this way, *miR-203* may act as a novel molecular target in bone cancer treatments. Furthermore, analyses conducted on rat BMSCs suggested that miR-203 is highly engaged in downregulation of phosphatidylinositol 3-kinase (PI3K) expression. Hence, it may decrease the PI3K/Akt signaling pathway and impair the viability of BMSCs, an extremely important population of progenitor cells ensuring proper bone metabolism and regeneration.^57^

Nevertheless, bearing in mind the dual and complex nature of *miR-203* in osteogenic differentiation, as well as its significant impact on the serious progression of metabolic diseases and development of various neoplasms, further in-depth analyses targeted on *miR-203* must be conducted.

#### miR-223

Moran-Moguel et al.^58^ proved that overexpression of *miR-223* significantly inhibits osteoclastogenesis in osteoporosis patients. *miR-223* promotes osteoblast differentiation of murine MC3T3-E1 cells by regulation of HDAC2 (histone deacetylase 2). HDAC2 acts as a negative regulator of osteogenesis.^59^ However, *miR-223* is additionally engaged in osteoclast differentiation by promotion of CSFR1/M-CSFR expression.^18^ On account of the dual effect in stimulating osteoclast differentiation and inhibiting osteoblast differentiation,^60^ the role of *miR-223* as a novel therapeutic target should be further analyzed. In addition, it has been shown that *miR-223* can prevent joint destruction in RA patients.^58^ *miR-223* is downregulated in serum collected from RA patients, but it is significantly upregulated in patients with anti-TNF therapy.^61^^,^^62^ When it comes to bone neoplasms, *miR-223* could be a novel pharmacological marker of OS, due to inhibition of metastasis progression.^63^^,^^64^ It was found that *miR-223* decreased the expression of PARP1, CtIP, and Pso4, which are significant components of alternative non-homologous end joining (aNHEJ). In most cells, the high level of *miR-223* represses aNHEJ, decreasing the risk of chromosomal translocation and reducing the probability of the development of malignancy.^65^ However, *miR-223* represents a crucial component of multiple myeloma development. It was shown that the *miR-223* transcript level was upregulated in huBMSCs delivered from multiple myeloma patients. This could be a result of a senescence-like state that is induced by activation of stromal cells by multiple myeloma cells. Moreover, *miR-223* seems to regulate important tumor-supportive cytokines, such as VEGF and IL-6.^66^

#### lncDANCR

The last evidence suggested that *DANCR* is one of the vital factors involved in the process of cell differentiation. It plays a crucial role in osteogenic differentiation of various types of cells, including stem cells. Furthermore, its contribution to the onset and development of osteoporosis is increasingly recognized. The downregulation of *DANCR* promotes the osteogenic differentiation of human periodontal ligament stem cells and human fetal osteoblastic cells. It has been shown that high expression of *DANCR* suppresses the differentiation of human dental pulp cells (hDPCs) by the Wnt/β-catenin signaling pathway into odontoblast-like cells. Moreover, upregulation of the DANCR transcript level blocked mineralized nodule formation and the expression of crucial odontoblast markers, such as *DMP-1* and *DSPP*.^67^ Additionally, *DANCR* knockdown enhances the levels of mRNA expression of osteogenic marker genes and mineralized matrix deposition in huBMSCs.^24^^,^^68^ Silva et al.^69^ have demonstrated that *DANCR* is overexpressed in monocytes in osteoporosis. Importantly, research has shown that media delivered from monocytic cell cultures, which have overexpressed *DANCR*, are characterized by increasing bone-resorbing activity in mouse bone cultures. The high level of this non-coding RNA is related to a significant and sudden increase of TNF-α expression, which is one of the most important inflammatory markers. The high expression of TNF-α is predominantly associated with osteoclast differentiation during osteoporosis progression. Furthermore, *DANCR* promotes RANKL-induced osteoclast formation, which also affects the development of osteoporosis.^69^ TNF-α is additionally strongly correlated with the development of RA, and it is called the “top of a pro-inflammatory cascade,” which means that this molecule plays a crucial role in the cytokine network of RA.^70^ Inhibition of *DANCR* expression, for example using antisense molecules blocking the *DANCR* expression, may yield satisfactory results in osteoporosis, as well as RA therapy. Importantly, note that *lncDANCR* is also highly engaged in OS cell proliferation.^71^ It binds *miR-33a-5p*, which leads to upregulated expression of AXL (AXL receptor tyrosine kinase). AXL is abnormally expressed in OS patients, regulates tumor cell self-renewal, and indicates a poor prognosis. Additionally, due to AXL upregulation, it enhances expression of proteins in the AXL-Act pathway.^71^ This signaling pathway regulates colony formation and EMT of the cancer stem cells (CSCs). On account of these facts, it seems to be a key molecule in many bone pathological pathways, including bone cancers.

#### lncTUG1

*TUG1* is an evolutionary conserved and common lncRNA present in various osteogenically induced MSCs, such as PDLSCs (periodontal ligament stem cells) or TPSCs (tendon stem/progenitor cells).^72^^,^^73^ It is considered to be a key factor facilitating the osteogenic differentiation of progenitor cells. It was shown that *TUG1* positively regulates *Runx2* expression by sponging the *miR-204-5p*. Therefore, one of the therapeutic strategies for the treatment of fractures in osteoporotic patients included a combination of pro-*TUG1* and anti-*miR-204* therapy at the same time, as a novel bone recovery approach.^24^ Sacchetti et al.^74^ have proved the validity of the *miR-204-Runx2* axis, as well as the *miR-211-Runx2* axis, in osteoporosis progression. Investigations carried out on murine MSCs indicated that enforced expression of *miR-204* inhibited osteogenesis and rescue adipogenesis of the cells. The intrinsic properties of MSCs are significantly altered in postmenopausal osteoporotic patients. They are characterized by poor osteogenic capability and increased adipogenic abilities. It is also known that osteoporosis and obesity often occur together.^75^ Therefore, future treatment methods of osteoporosis should be focused not only on elevation of the osteogenic abilities of MSCs, but on their inhibition of adipogenesis as well. Similarly to *miR-204*, the *miR-211* molecule is also negatively associated with Runx2 expression.^74^ The high levels of these markers are associated with low Runx2 level and dysregulated osteogenic processes. However, *TUG1* is abnormally overexpressed in OS cells, which pathogenically upregulates the *Runx2* transcript level and promotes the development of OS.^24^^,^^76^ Li et al.^77^ indicated that the overexpression of *TUG1* is associated with *miR-132/SOX4* axis dysregulation. Lowered expression of *miR-132-3p* is associated with *TUG1* overexpression, and this has a great impact on *SOX4* upregulation and OS progression. The knockdown of *TUG1* also markedly inhibits the expression of the MET and phosphorylated (p-)AKT signaling pathway, which is revealed by increased apoptosis rate and cell growth suppression in OS cell lines Saos-2 and MG-63. Additionally, the inhibition of *TUG1* expression significantly reduces the cisplatin resistance of these OS lines.^78^ Furthermore, *TUG1* is associated with poor prognosis for osteoarthritis patients, and the elevated expression of this lncRNA promotes osteoarthritis-induced degradation of chondrocyte extracellular matrix via the *miR-195/MMP-1*3 axis.^79^

#### lncMALAT1

The lncRNA *MALAT1* is a molecule predominantly considered to be an important amplifier of osteogenic differentiation of cells. *MALAT1* functions as a sponge molecule of *miR-204-5p* and upregulates the expression of *Smad4* (mothers against decapentaplegic homolog 4). The same sponging abilities against the *miR-204-5p* molecule are shown by *lncTUG1*.^24^ Smad4 activation promotes the expression of ALP and Ocl, considered to be essential osteogenic markers. Thus, *MALAT1* promotes bone formation and mineralization. This way, another alternative OP treatment could be based on pro-*lncMALAT1* and anti-*miR-204-5p* therapy.^4^ Moreover, *MALAT1* sponges *miR-30* and promotes osteoblast differentiation of ASCs (adipose tissue-derived MSCs) by significant promotion of Runx2 expression.^80^ Interestingly, *MALAT1* is responsible for downregulating *miR-124* expression, therefore affecting ALP, Runx2, and Opn levels and finally promoting osteogenesis of MSCs.^81^ The coupling mechanism between *MALAT1* and *miR-124* could be a remarkable and efficient direction for future osteoporosis therapy. Furthermore, *MALAT1* expression was proven to be reduced in synovial tissues of RA patients.^82^ Li et al.^82^ indicated that knockdown of *MALAT1* could stimulate the expression of pro-inflammatory cytokines, including IL-6, IL-10, and TNF-α. *MALAT1* could also suppress the expression of *CTNNB1* and modulate the Wnt signaling pathway. These findings suggest an inhibitory effect of *MALAT1* on the proliferation and inflammation of FLSs, which participate in the pathogenesis of RA, by inhibiting the Wnt pathway. Thus, *MALAT1* is suggested to be a perfect candidate for an OP and RA therapeutic target.

In contrast, in cartilage tissues collected from healthy and osteoarthritis patients, *MALAT1* was shown to be significantly upregulated. Moreover, *MALAT1* inhibits *miR-150-5p* expression, which has a great impact on elevated AKT3 expression. Thus, *MALAT1* is responsible for cartilage cells apoptosis, extracellular matrix degradation, and osteoarthritis development via the *miR-150-5p/AKT3* axis.^83^ However, it was additionally proven that *MALAT1* promotes the creation of metastases in OS patients.^84^ Upregulation of this molecule is associated with a high expression level of *SOX4* or activation of the Wnt/β-catenin signaling pathway.^85^^,^^86^

#### lncHOTAIR

*HOTAIR* is considered to be a significant diagnostic marker for many neoplasms, including breast cancer, cervical cancer, or colorectal cancer.^87^, ^88^, ^89^ Moreover, *HOTAIR* is considered to be one of the first tumor-related lncRNAs to have been discovered. It is associated with metastasis development and poor patient prognoses. In OS, *HOTAIR* was detected to be upregulated and coupled with *P53* expression. This indicates *HOTAIR* involvement in the P53-mediated apoptosis pathway in OS cells.^90^ It has been proved that *HOTAIR* promotes the proliferation and invasion of OS cells by the AKT/mTOR signaling pathway.^91^ However, it is also involved in bone regeneration from MSCs.^4^ *HOTAIR* reduces the expression of *miR-17-5p* and elevates the *Smad7* transcript level at the same time, and thus *Smad7* is a target of *miR-17-5p*. *Smad7* is an important factor that reduces osteogenic potential of the bone. Therefore, the knockdown of *HOTAIR* significantly upregulates the expression of *miR-17-5p*, suppresses *Smad7* activity, and finally increases the transcript levels of *Runx2*, collagen 1, and *ALP*.^4^^,^^92^ Furthermore, *HOTAIR* is considered to be an important factor in alleviation of RA. It was noted to be downregulated in lipopolysaccharide (LPS)-treated chondrocytes and RA mice. However, the transfection of *HOTAIR* increased cell proliferation and inhibited inflammation in RA mice. It can play a protective role in RA by regulation of the NF-κB signaling pathway. *HOTAIR* reduces the expression of *miR-138*, which activates HDAC4/PGRN or HDAC4/NF-κB signaling.^93^ It also inhibits the *P65*, *Il-1β*, and *TNF-α* transcripts,^94^ which participate in the development of RA. In contrast, it has been shown that *HOTAIR* is upregulated in osteoarthritis patients and indicates elevated expression of MMPs, as well as chondrocyte apoptosis. Therefore, the silencing of lncRNA *HOTAIR* could result in better prognoses for osteoarthritis patients.^95^

Table 1 summarizes the involvement of selected non-protein-coding RNAs in the maintenance of bone homeostasis. Their functionality in the course of osteogenesis and tumorigenesis is summarized. Additionally, we mention their targets and examined cell lines (Table 1).

**Table 1:** List of Selected Non-coding RNAs, Their Functions, and Targets

ncRNA	Impact on Osteogenesis	Role in Tumorigenesis	Examined Cell Lines	Targets	References
miR-17-5p	downregulation	oncogene	huASCs, MC3T3-E1, MG-63, U-2 OS, Saos-2, 143B	BRCC2, BMP-2, SMAD7, Wnt/β-catenin signaling	100, 101, 102, 103, 104
miR-21	upregulation downregulation (?)	oncogene	huBMSCs, MC3T3-E1, 4B12, MG-63, U-2 OS, Saos-2, HOS, 143B	SMAD family proteins, RUNX2, OCN, OPN, OCL, COLL-1, MMP-9, OPG, RANKL, RANK, IL-6, IL-8	^25^^,^^27^^,^^29^^,^^30^^,^^105^
miR-124	downregulation	suppressor gene	huBMSCs, MG-63	TGF-β family proteins, DLX family proteins, OSX, CDK2, MPC-1	^26^^,^^41^^,^^43^, ^44^, ^45^
miR-149	downregulation	suppressor gene	raBMSCs, MG-63, U-2 OS, Saos-2, HOS, 143B	ERK/MAPK signaling, SDF-1, PI3K/AKT pathway	106, 107, 108
miR-203	upregulation downregulation	suppressor gene	huBMSCs, huH226, MG-63, U-2 OS, Saos-2	RUNX2, DLX5, MMP-1, SMAD3, TGF-β family proteins, IL-6, RAB22A, TBK1	^47^^,^^49^^,^^51^^,^^54^, ^55^, ^56^
miR-223	upregulation downregulation	suppressor gene	MC3T3-E1, huBMSCs, 143B, U-2 OS	HDAC2, CSFR1/M-CSFR, CDH6	^18^^,^^58^^,^^59^^,^^63^^,^^109^
lncDANCR	downregulation	oncogene	huBMSCs, human monocytes, 143B	TNF-α, RANKL, miR-33a-5p, miR-216a-5p/SOX5	^69^^,^^70^^,^^110^
lncTUG1	upregulation	oncogene	huPDLSCs, huTPSCs, huBMSCs, MG-63, U-2 OS	miR-204, miR-211, miR-132/ SOX4, RUNX2,	^24^^,^^74^^,^^77^
lncMALAT1	upregulation	oncogene	huASCs, huFLSs huFOB1.19, MG-63, U-2 OS, Saos-2	miR-204, SMAD4, miR-30, miR-124, IL-6, IL-10, TNF-α, CTNNB1	^4^^,^^81^^,^^82^
lncHOTAIR	downregulation	oncogene	huBMSCs, huAVICs, MG-63	miR-138, miR-204, miR17-5p/SMAD7 axis, Wnt/β-catenin signaling, NF-κB signaling	^4^^,^^92^^,^^94^^,^^111^
lncH19	upregulation	oncogene	raBMSCs, raEMSCs, MG-63, U-2 OS, Saos-2, HOB	Wnt/β-catenin signaling, miR-138, miR-149/SDF-1 axis	^107^^,^^112^, ^113^, ^114^, ^115^

ERK, extracellular signal-regulated kinase; MAPK, mitogen-activated protein kinase; AVIC, aortic valve interstitial cell.

### Crosstalk between miRNAs and lncRNAs for Proper Bone Homeostasis

Non-coding RNAs are emerging as critical regulators of processes associated with bone metabolism, able to modulate complex cellular processes. Both miRNAs and lncRNAs act as fine-tuning molecules playing a crucial role in governing the expression of bone-related genes. It has been reported that lncRNAs are species-specific regulators of various metabolic processes. They may function as competing endogenous RNAs (ceRNAs) that can interact with mRNAs by competitively binding their common miRNAs. In bone tissue, non-coding RNAs are responsible for processes that are crucial for proper musculoskeletal system functions, such as bone turnover or tissue regeneration. However, minor shortcomings in the functionality of expanded networks between non-coding RNAs and mRNAs may contribute to pathological changes of the tissue structure. In addition, miRNAs and lncRNAs regulate the proliferation and differentiation of bone-forming and bone-resorbing cells.^96^

*lncMALAT1* serves as a sponge for *miR-30* and *miR-124* and elevates the differentiation of osteoblasts. In addition, *MALAT1* inhibits the activity of *miR-204* and thus increases the *Smad4* level, which finally leads to proper osteoblast functionality.^4^^,^^80^^,^^81^ Furthermore, *miR-204* as well as *miR-211* are blocked by *lncTUG1*.^24^^,^^74^ Therefore, *TUG1* serves as a positive factor in osteoblast activity and proper bone mineralization. *lncHOTAIR* sponges *miR-17-5p*, which leads to increased *Smad7* transcript levels. This signaling pathway results in downregulated osteogenic potential of the bone.^4^ *lncDANCR* positively regulates osteoclast differentiation by upregulating *TNF-α*. Furthermore, *DANCR* elevates the RANKL/OPG ratio via sponging *OPG* and downregulating *RANKL*. It leads to upregulated osteoclast activity and facilitates bone resorption.^69^ Moreover, *miR-21* also elevates the RANKL/OPG ratio; however, it is also considered to be a crucial factor that positively affects osteoblast activity and enhances the mineralization of bone tissue.^25^

Despite the critical driving force of the non-coding RNA network in bone-dependent disease progression, attention is more often paid to single nucleotide polymorphism (SNP), which is a common genetic variation. This is considered to be an important factor that modulates susceptibility to serious diseases, e.g., cancers.^97^ For instance, it has been proven that the SNP of *miR-124a* significantly affects the risk and determines the prognosis of OS.^98^ Moreover, a single miRNA can differ at the 5′ or 3′ terminus by minor changes. This can result in the formation of isoform of specific miRNA (iso-miR). The numerous variants of single miRNAs could be associated with disease progression; however, more in-depth studies are required to explore iso-miRs as future therapeutic targets.^99^

### Conclusions

It is estimated that around 70%–90% of mammalian genomes are transcribed, but the vast majority of transcripts do not code proteins. The rapid development of molecular biology techniques, especially next-generation sequencing technologies such as RNA sequencing (RNA-seq), makes it possible to verify that non-coding transcripts are not only junk or “transcriptional noise,” but also essential regulators of gene expression. Non-coding RNAs are engaged in many important biological processes, providing a unique regulatory mechanism for genes coding proteins, including morphogens and growth factors, which assure the proper development as well as homeostasis of an organism. There is a great need to explore this enormous world of functional classes of non-coding RNAs. In our opinion, special attention should be devoted to the identification and analysis of crosstalk between miRNAs, lncRNAs, and putative target genes. Evaluation of this functional network is crucial, notably in the view of better understanding of the molecular basis for the pathogenesis of lifestyle diseases, such as osteoporosis and RA. The analysis may also provide novel panels of biomarkers showing prognostic and diagnostic potential for bone-related diseases. Importantly, analysis of miRNA-lncRNA-mRNA crosstalk and networks may disclose new targets and allow the design of better therapies and therapeutic approaches, such as personalized medicine for bone disorders.

In this review, we have presented current knowledge related to the biology and function of miRNAs and lncRNAs that are involved in the process of osteogenesis and may find application as novel biomarkers for bone-related diseases. Several biotypes of non-coding RNAs, including miRNAs and lncRNAs, were identified in the cargo of extracellular vesicles released to the biological fluids. Thus, non-coding RNAs can act locally (in a paracrine and autocrine manner), as well as on adjacent cells. This is an important biological aspect that allows us to describe full panels of biomarkers, with paramount clinical importance in terms of personalized regenerative medicine for bone. It was shown that *MALAT1* and *TUG1* can serve as vital therapeutic targets, especially for osteoporosis patients. Due to affecting *miR-30, miR-124, miR-204*, and *miR-211*, presented lncRNAs are significantly engaged in the process of proper bone mineralization. Moreover, *HOTAIR*, by affecting *miR-17-5p* expression, may serve as a remarkable diagnostic and prognostic factor for tumor development and osteoporosis progression. Additionally, *DANCR* can act as another essential therapeutic target, especially for the treatment of bone metabolic diseases. We strongly believe that the presented information on selected non-protein-coding RNA molecules will serve as an important impetus for preclinical investigations.

## Author Contributions

Concept of the Review, M.S. and AS; Graphical Work, M.S.; Writing – Original Draft, M.S. and A.S.; Writing – Review & Editing, M.S., A.S., and K.M. All authors have read and agreed to the published version of the manuscript.

## Conflicts of Interest

The authors declare no competing interests.
